# Application of Walkability Index for Older Adults’ Health in the Brazilian Context: The Case of Vitória-ES, Brazil

**DOI:** 10.3390/ijerph19031483

**Published:** 2022-01-28

**Authors:** Daniella do Amaral Mello Bonatto, Fernando Brandão Alves

**Affiliations:** 1 Department of Architecture and Urbanism, Federal University of Espírito Santo (UFES), Vitória 29055-310, Brazil; 2 CITTA, Faculty of Engineering, University of Porto, 4200-465 Porto, Portugal

**Keywords:** walkability index, older adults’ health, older persons, WIEH, public space, Vitória

## Abstract

This study follows up on the article ‘Walkability Index for Elderly Health: A Proposal’, published in 2020, as well its validation in the Historic Center of Porto, published in 2021. The 2020 article presented the theoretical and methodological bases relating qualities of public spaces, the walkability of older adults, and the direct benefits on health. The 2021 article validated the applicability of the index in the Historic Center of Porto, Portugal. Both articles incorporated the factor ‘slope’, solving a criticism evidenced in related literature about the slope being ignored in older adults’ walking conditions studies. The present study, however, aims to validate the conceptual design of the Walkability Index for Elderly Health (WIEH) in the Brazilian context at the historic center of Vitória. The methodology included the analysis and evaluation of public spaces regarding the pedestrian network—urban fabric, urban scene, and safety—and the presence of slopes and/or stairways. Subsequently, these spaces were classified according to the WIEH, ascertaining their level of adequacy for older adults’ walkability. The results show that paths friendly to older adults are rare and that the problems encountered focus primarily on the low quality of the pedestrian network and not on the existence of slopes and stairways.

## 1. Introduction

Due to the irreversible concentration of population in urban centers and the acceleration of aging, cities are urged to adapt towards promoting and ensuring active aging [1], which greatly depends on urban structure and the physical conditions of space for the mobility of people; to older adults, especially, walking is considered an important form of locomotion, given its direct health benefits [2]. Walking deserves greater prominence because it is available to everyone and does not require special learning skills, besides being an appropriate way to promote physical activity [3].

The report ‘World Population Prospects 2019: Highlights’ [4] pointed out that all regions and countries are experiencing population aging, highlighting its faster rate in Latin America and the Caribbean, with the population aged 65 and older expected to jump from 8.7% in 2019 to 12.0% in 2030 (a 50% increase in just 11 years); twice as many older people are expected in 2050. The same report indicated that global life expectancy will increase from 72.6 years in 2019 to 77.1 in 2050, when more than twice as many people will be aged 65 and over as children under 5. Brazil already faces the challenges of a rapidly aging country [3]. The World Health Organization (WHO) has projected that, by 2032, the country may be considered old, with 32.5 million people aged 65 years or older out of a total of more than 226 million Brazilians [5]. This population trend points to the need for greater care regarding human aging and health. Older adults’ mobility, especially walkability, is fundamental to active aging [1], constituting a determinant of individual health, access to urban services, and social interaction. The guide for age-friendly cities [6] reinforces the role that the urban environment and mobility conditions have in human aging; they can become either an invitation to walk or a risk factor when considering this activity, as well as a determinant of travel choices.

There are many studies in the available literature that evaluate spatial characteristics and their relationship with walkability [7,8,9]. However, the relationship between slopes in the urban fabric and walking are poorly studied within the field of transportation [10] and mobility; furthermore, there is a gap regarding the presence of stairways in walkability studies [11]. In the health field, especially gerontology, there are also several studies on the relationship between physical activity and health in older adults. However, the relationship between walkability in each environment and its direct potential for the health of older adults is still sparsely researched [7]. This approach was used in the project titled Mobi-Age Promotion of Urban Mobility for Aged Populations [12], which gave rise to the development of the Walkability Index for Elderly Health—WIEH [7]. The relevance of the WIEH lies in incorporating slopes and stairs into physical audits but also in relating walkability conditions to the potential for direct health benefits in older adults.

This paper adopts the WIEH and applies it to the historic central area of the city of Vitória, Brazil, as a follow-up to its application in the Historic Center of Porto [13], with the main goal of validating the referred index in the Brazilian urban context. The Brazilian context is considerably different from that found in European cities, in terms of the quality of urban infrastructure for walking, as well as in terms of public space management-namely sidewalks. The scientific and applicative contribution of this paper remains in its conceptual and methodological approach—linking physical audits and the potential for health—and in providing a possibility to compare different urban contexts relevant to the field of urban studies and to the discussion of older adults’ walkability in Brazilian cities. The choice for Vitória lies in the fact that it is one of the Brazilian cities with the highest life expectancy, having a population percentage of older adults above the national average, besides the fact that it is recurrently pointed out as a good city to grow old in.

## 2. Aging, Walkability, and Health

Although the significant increase in life expectancy is a contemporary achievement, it cannot be said that current and future generations will experience a healthier and better aging process [14], especially if cities do not adapt their spatial support and public policies to the requisite quality of life. The creation and maintenance of favorable contexts for healthy and active aging are key to prolonging the physical and mental conditions of individuals in order to delay and reduce the loss of capabilities, especially in relation to mobility. The community within which a person lives plays a decisive role in producing well-being when aging or, on the contrary, producing an old age marked by exclusion [14,15].

The environments that enable quality of life are those that promote the longevity and autonomy of older adults, despite the increasing physical limitations imposed by aging [6,14,16,17,18]. Factors extrinsic to the individual affect how individuals and populations age [1,14]. There is a need to ‘redesign urban areas to increase attractiveness and well-being’ and ‘invest in improving walkability in urban areas’ [19]. In 2005, the WHO launched the Age-Friendly Cities initiative [6], fostering the formation of a Global Network for Age-Friendly Cities and Communities to promote the planning, measurement, and monitoring of age-friendly urban physical environments and public policies [6,20,21].

The act of walking positively affects the body and brain [22] and constitutes an important physical exercise. Regular physical activity is a factor in preventing and controlling non-communicable diseases such as cardiovascular diseases, type 2 diabetes, and breast and colon cancer [23]. It also improves mental health, delays dementia, contributes to weight control, and promotes a general sense of well-being. The WHO (2020) [23] recommends regular physical activity—from 150 to 300 min per week of moderate to intense activity—to senior people.

A recent meta-analysis [24] presented more accurate data on the relation between physical activity-even in its lightest form-and decreased mortality risk. According to the authors, physical activity baselines usually rely on information provided by the individual and are subject to error and underestimation in the magnitude of correlations between physical activity and mortality, especially for light physical activity. The meta-analysis collected only objective data from studies using an accelerometer—a device with a movement sensor that records the volume and intensity of physical effort. These studies demonstrated that even light exercise is substantially linked to a reduced risk of death. The meta-analysis, in particular, emphasized that this relation is twice the magnitude of what is found by studies based on information provided by the participants [24]. Thus, light physical activity contributes more than previously known to death risk reduction—each step taken helps save a life [25]. These data reinforce the importance of including walking in daily activities, especially when fighting against sedentary lifestyles; a person who stops being sedentary decreases their risk of death from cardiovascular disease by 40% [3].

Besides the fact that a sedentary lifestyle is related to higher mortality risk and that physical activity prevents chronic disease [1,23], a person’s physical status is known to influence vulnerability to COVID-19; namely, a better cardiorespiratory condition may constitute an immunological protective factor in patients who contract SARS-CoV-2 [26]. Since physical activity enhances the immune response to infections [27], it is expected to reduce the risk of a SARS-CoV-2 infection and its severity [26]. For all this, exercise and sport practice should be regarded as essential activities, especially in pandemic times [28].

Despite these known health benefits, adherence to physical activity is low. A study [29] of over 380,000 adults in the United States revealed that only 18% of adults aged 65–74 years and 15% of those over 75 years meet the minimum recommendations for cardiovascular exercise and muscle strength set by the WHO. This low adherence to physical activities reinforces the need for an urban environment that promotes them, especially walking, whose importance for older adults’ health was highlighted in a Brazilian government booklet of basic care for the older adults (*Caderno de Atenção Básica ao Idoso*), published by the Ministry of Health [3].

Older adults who live in areas with physical barriers tend to go out less and become more prone to isolation, depression, physical unpreparedness, and mobility problems [1]. Older adults have very specific spatial needs that need to be addressed [30]. Hazards in the physical environment can cause disabling injuries, commonly linked to falls and automobile accidents [1]. Older adults’ falls are associated with high rates of morbidity and mortality, reduced functional capacity, and early institutionalization [3]. Approximately 30% of older adults in Brazil fall each year; this rate increases to 40% among those over 80 years old and to 50% among those living in long-term institutions [21]. Some of the main causes leading to older adults’ falls in urban space include inadequate lighting, slippery surfaces, potholes and irregularities in sidewalk flooring, inadequately sized steps, and obstacles [31]. A longitudinal study of Brazilian older adults’ health, Estudo Longitudinal da Saúde dos Idosos Brasileiros (ILSI-Brazil), which included 9412 people in 70 cities, showed that 56.1% of all older adults are afraid of falling while out on the street due to the quality of sidewalks, 48.9% are afraid of crossing the street, and 35.5% feel their neighborhood is unsafe [32]. It should be noted that exercise can reduce the rate of older adults falls by 23% [23].

Another walkability problem concerns road safety and traffic accidents. Brazilian average pedestrian death rate per 100 thousand inhabitants affects mostly the 50–59 and 60 or above age groups [33]. In the state of São Paulo, 33% of deaths by pedestrian collisions between 2015 and June 2021 were of older adults [34]. In the city of Vitória, between 2015 and 2019, there were 1266 pedestrian collisions, 32 of them within the area of this study [35]. In 2018, among all traffic deaths, 38% were pedestrians [36]. The cited data reinforce the need for the urban environment to reduce injury risks and promote physical activity and independence for older adults [37]. A welcoming environment contributes to interaction in public spaces, and the presence of green areas encourages walking, reduces stress, improves mood, and facilitates social encounters [37]. Therefore, the human dimension [38] is a key factor that must be promoted in the planning and design of cities in order to make them lively, safe, sustainable, and healthy. In this scenario, active mobility plays a key role [39] and should be encouraged through an environment that prioritizes people, accessibility, and a lack of barriers to walking.

A booklet on aging and older adults’ health (*Caderno Envelhecimento e Saúde da Pessoa Idosa*), published by the Brazilian Ministry of Health [3], recommends walking in flat places, avoiding extreme temperatures (too hot or too cold), and opting for low humidity. The existence of green areas and urban afforestation is determinant for thermal comfort and walkability, especially in cities with humid tropical climates; this is the case in Vitória, whose average temperature registers 34.4 °C in summer and 24.4 °C in winter, with surface temperature in urbanized areas ranges from 18 to 49 °C [40] and the existence of several heat islands both in summer and winter [41]. Thermal comfort has also been addressed in studies pertaining to vegetation and health. A book on urban life and health (*Vida Urbana e Saúde* [42]) demonstrates how climate extremes can kill, as extreme heat increases the risk of heart attacks and strokes, which potentially lead to death. Urban afforestation is considered in several walkability studies [7,9,43,44].

Other aspects of the environment are considered determinant to walkability, such as neighborhoods with mixed uses and essential commerce and services, sufficient urban furniture, or pleasant areas known for active facades and safety [38,43,45], especially in what concerns older adults [1,6,7,9,36,41,46,47]. On the other hand, the absence of support for urban furniture—mainly the lack of benches, drinking fountains, and accessible public toilets—makes it difficult for older adults to stay outdoors [6]. In Brazil, 30% of the non-institutionalized older adult population suffers from urinary incontinence [3], further increasing the need for public toilets.

Another particular aspect that directly affects the mobility of older adults is topography [37]. As previously mentioned, it is necessary to investigate slopes in studies of older adults’ walkability [7,9,44]. The effect of slopes on the human body is well demonstrated in the field of health and exercise physiology [7,10]. However, the relationship between terrain slopes and walking activity is poorly studied in the field of transportation engineering [10] and mobility, yet there is evidence of the negative influence that slopes exert on attractiveness for walking and route choice. One more research gap, in addition to slopes, is the lack of stairways among environment factors when researching the relation between walkability and physical activity, as pointed out by a systematic review [11]. This work observed that, although the presence of stairways is singled out by older adults as a challenge to walking, works that include stairways among the elements to be audited are very rare.

In the Brazilian context, cities are not suitable for walking [48,49,50,51]. A survey conducted in the 27 capital cities [49] on the conditions of sidewalks directly maintained by the government indicated inadequacy for walking. The very poor evaluations highlighted irregularities in the floor, problems in crossing lanes, and lack of accessibility ramps, all characteristics that lead to falls and pedestrian accidents. The responsibility of building and maintaining sidewalks is delegated to lot owners, and, thus, walking is not visualized as something public nor properly considered in road planning and design, which remain focused on the automobile [48]. This situation contributes to the poor walkability conditions of Brazilian cities. Another aspect highlighted is the lack of municipal data on sidewalks, a symptom of the low priority attributed to walking mobility [48]. This lack of priority makes it difficult to establish an adequate pedestrian network and thus greatly penalizes older adults.

An age-friendly city should feature well-maintained green spaces; accessible toilets; well-maintained public benches at regular intervals; pedestrian-friendly and obstacle-free sidewalks with level, non-slip surfaces and sufficient width to accommodate a wheelchair; low curb corners to facilitate the transition to the street; and safe crosswalks and pedestrian priority [6]. It is also of note that an unsuitable walking environment can be reversed, as demonstrated by a study [52] that identified a change in population behavior following redevelopment in the ancient city of Zhangzhou. The study measured an increase in walking distances and walking frequency after the changes in the urban environment: implementation of green spaces; insertion of furniture; restoration of historical open spaces; occupation of empty lots surrounding these open spaces, transforming the void into commercial buildings [52].

In order to more adequately guide the planning and design of urban space that is friendly to older adults, it is necessary to carry out field surveys, georeference them, and make assessments that determine the suitability of the urban environment for walkability. A more in-depth study of the physical conditions for older adults’ mobility and health, such as the one proposed by the WIEH, can reduce the knowledge gaps identified by the literature while providing friendlier routes for daily commuting.

## 3. Materials and Methods

**Figure 1:**
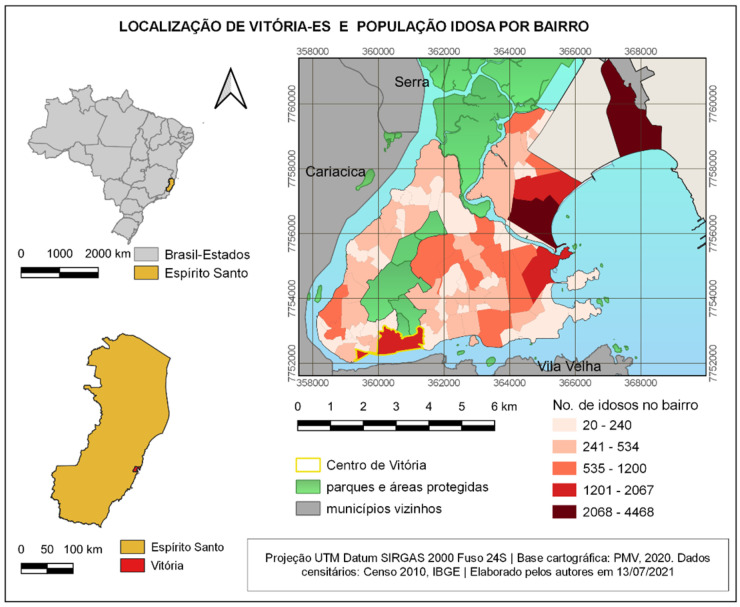
Location of Vitória-ES, Brazil, and older adults’ population per neighborhood. Source: created by the authors in 13 July 2021, based on GeoWeb/PMV data and cartography [54].

This study was implemented in the Brazilian city of Vitória, capital of the state of Espírito Santo, located on the country coast (20°19′09″ south latitude and 40°20′50″ west longitude). Most of its territory is within a fluviomarine island. It has an estimated population of 369,534 inhabitants, an extension of 96.53 km^2^, and a demographic density of 3338 hab/km^2^ [53]. The Metropolitan Region of Greater Vitória comprises seven municipalities. The neighborhood known as Center (Centro) is the 6th most dense in the municipality and the 4th in what concerns population aged 60 years or more. According to the 2010 Census (IBGE), in this neighborhood, 19.9% of the population are older adults (Figure 1), while the municipal average is 11.4% and the Brazilian average is 13%. According to the population projection made in 2018 by IBGE, the percentage of older adults in Brazil will double in the coming decades.

**Figure 2:**
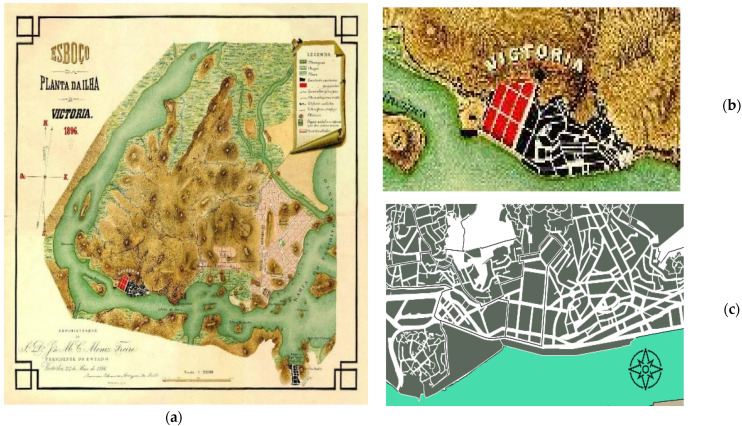
(**a**) Schematic plan of Vitória, from 1896 (black corresponds to its oldest occupation, red to the first embankments, and pink to the New *Arrabalde* Project for expansion; (**b**) Detail of the 1896 plan for the center of Vitória; (**c**) current morphology. Sources: (**a**,**b**): http://legado.vitoria.es.gov.br/baiadevitoria/ (accessed on 13 July 2021); (**c**): created by the authors, based on GeoWeb/PMV cartography [55].

Vitória has national historical importance as the third oldest capital city in Brazil, founded in 1551. It has great centrality in the state, and it receives a large amount of daily population flow due to its statal centrality and capacity for concentrating the Metropolitan Region employment opportunities, specialized services, and tourism. The preliminary layout of Vitória followed the Portuguese urbanistic tradition, presenting principles of military engineering [54]. The city expanded from a religious center and a port nucleus, with the layout of the founding nucleus still visible to this day (Figure 2).

**Figure 3:**
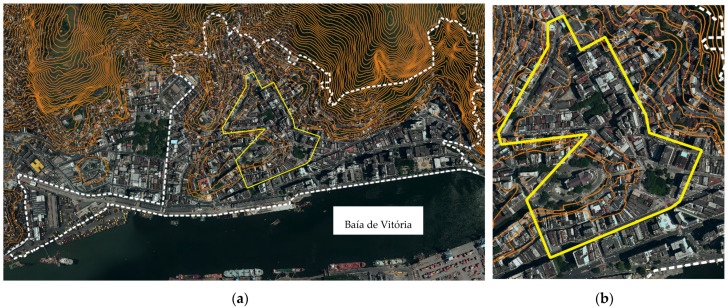
(**a**) Center neighborhood delineation (white dash); (**b**) highlighting the study area in the historic center of Vitória, Brazil (yellow). Source: created by the authors in July 2021, based on GeoWeb/PMV cartography [55].

The city had part of its occupation based on landfills, which explains its diverse urban morphology. The Center is divided in Cidade Alta and Esplanada Capixaba. Cidade Alta corresponds to the oldest urban formation, with steep slopes, irregular layout, some narrower streets, few pedestrian streets, and several stairways. The lower part, Esplanada Capixaba, is the result of successive landfills, with a more regular layout, longer blocks, and wider streets. The downtown area (Figure 3) is characterized by the presence of the port, the historic center, the seat of the State Government, several public facilities, stores, residences, food services, and nightlife. The region also has buildings listed as historical heritage, while some other buildings have garnered interest in their listing. However, the region lacks revitalization.

This paper applies the WIEH [7] to a section of the historic center of Vitória (Figure 3) in order to evaluate the walking conditions of older adults and validate this index in the Brazilian context.

The study area corresponds to 91 thousand m2 involving 20 blocks and just over 6706 m of sidewalks, divided into 126 survey sections: among them, there are 4 historical stairways. The dimensions of this area are approximately 400 m per 480 m. This choice took into consideration the literature review of the 2020 article [7], which pointed out the importance of locating primary services within a 400 m radius, and 500 m as the maximum comfortable walking length for older adults. The delimited area tried to encompass important urban equipment: a ‘Senior Citizens Center’, four squares including one with a ‘senior citizens gym’, supermarkets, bakeries, pharmacies, lottery shops, banks, clinical analysis laboratories, medical offices, gyms, a shopping mall, several neighborhood stores, restaurants, cafes, the Metropolitan Cathedral, theaters, and points of historical and tourist interest.

The study used municipal cartographic bases from GeoWeb (vitoria.es.gov.br, accessed on 14 September 2020). For the application of the WIEH, an extensive audit on foot, undertaken between January and March 2021, constituted the starting point. The study area was divided into sections, with each section of sidewalk being defined according to the intersections between the street axes through the georeferenced base of the municipality. Thus, some blocks have more than one stretch of sidewalk due to the existence of intersections in the route. The geographic data processing and the map creation made use of the free software QGIS, Version 3.10.11-A Coruña (https://qgis.org/en/site/, accessed on 14 September 2020).

## 4. WIEH Application in the Historic Center of Vitória

The application of the WIEH in the historic center of Vitória followed the methodological orientation of the aforementioned index [7]. It involved (i) the evaluation of pedestrian network quality (‘isw’), (ii) the integration of slopes and stairways, and (iii) the calculation of the WIEH based on ‘isw’ and ‘is’.

**Figure 4:**
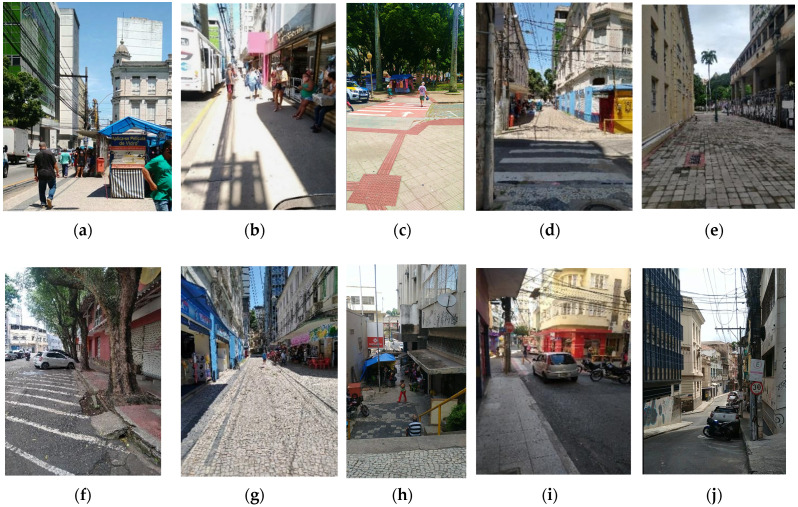
Photos of the study area in Vitória, Brazil, taken between January and March 2021 (**a**) Av. Jerônimo Monteiro; (**b**) Av. Jerônimo Monteiro, showing the absence of benches and people resting against the wall of the store; (**c**) passageway to Praça Costa Pereira; (**d**) passageway in front of R. João Aguirre; (**e**) Rua João Caetano, exclusively for pedestrians; (**f**) a corner of Praça Ubaldo Ramalhete Maia, with slope and crossing issues; (**g**) Rua João Aguirre, exclusively for pedestrians; (**h**) view of Rua Cerqueira Lima, exclusively for pedestrians, with stairway and street commerce in the background; (**i**) raised crosswalk at the intersection of Rua Prof. Baltazar and the pedestrian street Sete de Setembro; (**j**) stretch of Rua Duque de Caxias, with steps and a steep slope.

Figure 4 shows some of the walking spaces in the study area, where most of the roads have segregated lanes for vehicle and pedestrians. The study area only possesses 15 exclusive stretches for pedestrians (11.9%), with four stairways (3.2%)—namely, Maria Ortiz, São Diogo, Erothildes Resendo and Dionísio Rosendo—and eleven flat stretches (8.7%)—two stretches of Rua Sete de Setembro, plus João Caetano, Rua Carlos Gomes, Rua Cerqueira Lima, Rua da Alfândega, Rua Luiz Antônio, Rua João Aguire, Praça Dom Luis Escortegagna, Rua Senador Milton Campos, and a stretch next to Praça Ubaldo Ramalhete.

### 4.1. Physical Audit—Dimensions of the Pedestrian Network

This section presents the data audited in the field, organized according to the three dimensions determined by the WIEH (Table 1)—urban fabric, urban scene, and safety—and 11 variables.

#### 4.1.1. Urban Fabric

It was observed that there is rarely uniformity of conditions along each stretch, and there are even risky situations that can lead to stumbles and falls (Table 2).

Regarding the variable SE—sidewalk existence, the study area was well evaluated, having 95.2% of the stretches with sidewalks and only 6 stretches (4.8%) with some parts without a sidewalk.

For the evaluation of SW—sidewalk width, the same classification parameters of the WIEH Porto study were applied [13]. However, instead of computing the total width of the sidewalk, this work measured the critical width—that is, the smallest width found per pedestrian passage, in order to meet the Brazilian accessibility normative established in NBR 9050 [56]. The NBR 9050 divides the sidewalk into three bands of use: (a) a service band, which accommodates furniture, flowerbeds, trees, and lampposts or signaling; (b) a free band or sidewalk, exclusive for pedestrian circulation, which should be free of any obstacle, continuous along the block and have a minimum width of 1.20 m; (c) an access band, located next to the lot and applicable to sidewalks wider than 2.00 m, which can accommodate access ramps if authorized by the municipality. In the studied area, 31.7% of the evaluated stretches have a critical width of less than 1.20 m, and 14.3% of all stretches lack even 90 cm of passage. Another 34.9% present widths between 1.20 and 1.79 m, and 33.3% are wide sidewalks, with critical widths greater than 1.80 m (Table 3).

The variable TSI—traffic street intersection evaluates the continuity of pedestrian routes or the existence of intersections with car traffic, which makes sense in pedestrian areas or areas with pedestrian predominance, as is the case in many historic centers. In the historic center of Vitória, there are few exclusively pedestrian streets. Considering the Brazilian standard on accessibility [56], this work did not evaluate the number of intersections with traffic but rather how safe and accessible they were, since when adequate, these crossings do not impede safe walking. The Brazilian standard NBR 9050/2015 [56] establishes that crossings must either be elevated (prioritizing the pedestrian) or opt for a lowering of sidewalks according to the flow of pedestrians, with a slope that does not exceed 8.33% and a minimum width of 1.50 m. The ramp may not decrease the minimum width of 1.20 m of the free lane, nor may there be unevenness between the end of the sidewalk lowering and the roadbed.

That said, the evaluation of the TSI variable (Table 4) analyzed both ends of each crossing. Only those which had a ramp with an adequate slope at both ends and tactile floor according to the standard had a score of 3 (adequate crossing). In some cases, sidewalks were continuous between one stretch and the other; if they proved of good quality, without unevenness, a score of 3 was also computed. 

The evaluation of the EoS—existence of stairs (Table 5) classified as stairways any group of four or more steps, with a flight of stairs equivalent to eight steps (1.5 m of unevenness). Although the Center possesses several instances of unevenness and many stairways, only 11 stretches with stairs were observed in the studied area, five of them (4.0%) with several flights. Therefore, 91.3% of the stretches do not present this difficulty.

The evaluation of the variable EoO—existence of obstacles (Table 6) showed that only 46.0% of the stretches in the studied area are free of obstacles while noting the lower walking quality of the other 49 stretches (38.9%), some of which are problematic. When obstacles impeded the passage of the pedestrian or wheelchair user, it was evaluated with a score of 1. The obstacles observed included streetlamp posts, traffic signposts, wide or deep holes that demanded a detour, isolated steps that can cause falls and impede the passage of wheelchair users, and street vendor stalls.

**Figure 5:**
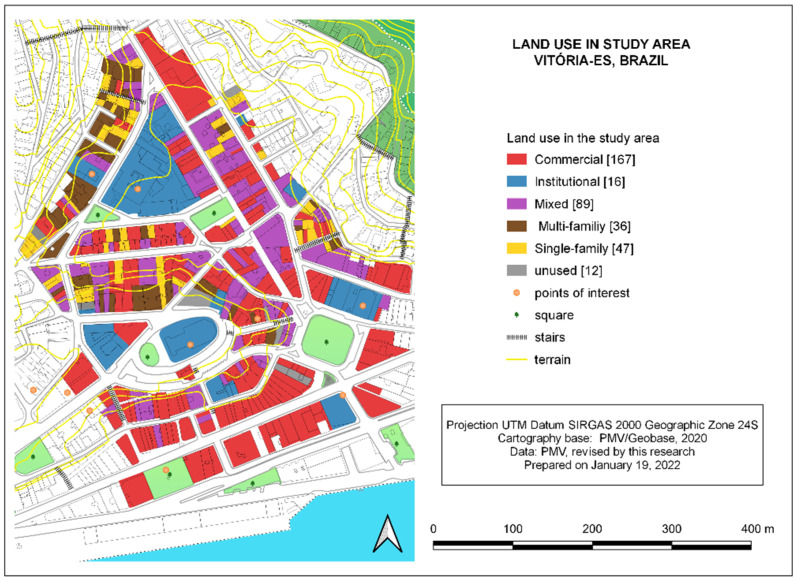
Land use in the study area, within the historic center of Vitória, Brazil. Source: created by the authors in July 2021, based on data and cartography from GeoWeb/PMV [54].

As for land use mix (LUM), the study area has a predominance of mixed use, standing out some points of special interest: The SESC Glória Cultural Centre (Centro Cultural SESC Glória), the Carlos Gomes Theatre (Teatro Carlos Gomes), the Metropolitan Cathedral (Catedral Metropolitana de Vitória), the Rosário Church (Igreja do Rosário), the Senior Citizens’ Centre (Centro de Convivência do Idoso), the Homero Massena Gallery (Galeria Homero Massena), the State Public Archive (Arquivo Público Estatal), and the colonial residences on José Marcelino Street, now the headquarters of the Institute of Historical Heritage (instituto do Património Histórico). Of the 20 blocks studied, only four (20%) are monofunctional, with commercial or institutional use. Four squares (Costa Pereira, Ubaldo Ramalhete, Irmã Josepha Hosanah and Dom Luiz Scortegagna) and 355 plots in use were computed—167 (47.0%) exclusively commercial, 89 (25.1%) mixed, 83 (23.4%) residential, and 16 (4.5%) of institutional use (Figure 5).

The study area presents a great diversity of commerce and services, with 64.3% of the stretches displaying mixed use (Table 7). It is worth noting, however, that 35.7% of the evaluated stretches have a monofunctional use, which is less desirable for urban vitality and, consequently, bad for walkability.

#### 4.1.2. Urban Scene

**Figure 6:**
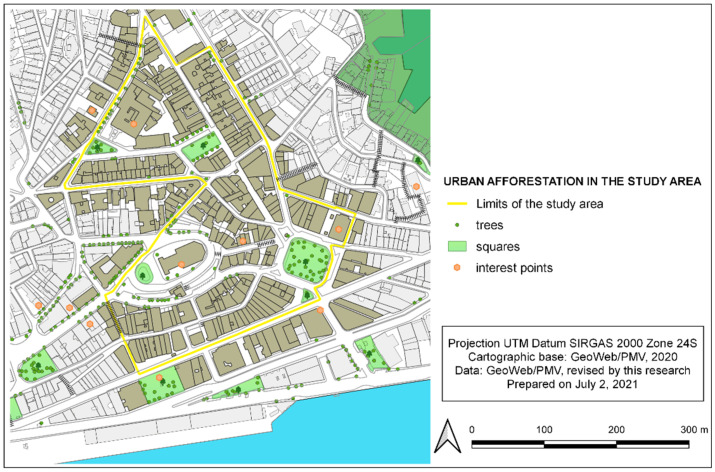
Urban afforestation in the study area, within the historic center of Vitória-ES, Brazil. Source: created by the authors in July 2021, based on data and cartography from GeoWeb/PMV [54].

The urban scene is the second composition dimension of ‘isw’ and concerns the existence of trees/vegetation and street furniture. The evaluation of the variable ETV—existence of trees/vegetation showed that most of the area under study (65% of the stretches evaluated) is sparse regarding urban forestation and landscaping (Table 8). Only 137 trees were computed in the studied area (Figure 6).

When evaluating the variable EUF—existence of urban furniture within the study area, only 13.7% of the sidewalk stretches possess benches, all of them located exclusively in the four squares (Table 9). Several bus stops can be observed, but only seven have shelter. Furthermore, no public restrooms or drinking fountains were found. It was observed that many squares or wider sidewalks had tables and chairs for private use, restricted to bars; in one of the stretches, this restricted-use furniture even strangled the passage of pedestrians.

#### 4.1.3. Safety

Safety is the third dimension of the ‘isw’ composition, pertaining to the quality of lighting and the presence of information to guide walking. SLQ—street light quality was evaluated by observing the existence of specific lighting for pedestrians (public or affixed to buildings) and by analyzing whether the street lighting met the sidewalks, being classified respectively as good or acceptable. The evaluation was conducted during the pandemic period when restrictions on the operation of activities made the environment less busy, which had the potential of skewing night surveys. Thus, the evaluation was predominantly done during the day without assessing luminance or confirming the existence of shadow points. Only flagrant cases of poor quality were rated (9.5%). Despite this context, only slightly over half (51.7%) of the evaluated stretches are classified as having good pedestrian lighting (Table 10).

The variable DIS—diversity of information signs (Table 11) showed a strong absence of pedestrian information (71.4%). Over 28% of the evaluated stretches have some signage to guide walking. The areas evaluated as having medium diversity of information possessed an indication of the street name and a bus stop (usually a simple sign, without information about the lines that serve the location). Only the areas evaluated as good presented some directional signs, usually related to tourist attractions and displayed on tall posts, favoring orientation for drivers. A few locations presented more information, such as a map of the region with elements of touristic interest and historical facts about the place where they were located. Therefore, general pedestrian orientation difficulties were observed within the historic center, especially for non-residents.

### 4.2. The Index of Space Walkability (‘isw’)

The Index of Space Walkability (‘isw’) was evaluated based on the preliminary analysis of each variable of the presented dimensions—urban fabric, urban scene, and safety. The weight of each variable is defined by the WIEH proposal [7]. In the application of ‘isw’ to the Brazilian city of Vitória, some weight adjustment was necessary in order to ensure greater participation of two priority variables—PSQ and ETV.

The first variable that had its weight increased was PSQ, which assesses the quality of sidewalk paving, a very delicate issue in Brazilian cities due to the diversity of people responsible for sidewalks within the same block (each lot owner is responsible for their stretch of sidewalk), giving rise to the great heterogeneity of design, maintenance, and quality. During the field survey, the pedestrian network was observed to possess great irregularity: differences in flooring, maintenance, presence of holes, and unevenness. Several complaints and reports of falls, especially among the older adults, were also received. All of this led to the proposal of increasing PSQ weight from 0.08 to 0.12. 

The second adjusted variable was the ETV, which evaluates the existence of vegetation and trees. Brazilian cities tend to be very hot in summer, especially coastal cities such as Vitória. Temperatures are high throughout the year, penalizing walkability in areas without urban trees, especially between 10 a.m. and 4 p.m. Although the sun sets early in Brazil, even in summer (around 6 pm), thermal comfort is restricted throughout most of the day, a fact aggravated by the early closing of business hours, usually at 6 pm. Therefore, ETV weight was proposed to increase from 0.08 to 0.10. 

Based on the identified necessities, and in order to balance the original equation, the variable DIS, which evaluates the diversity of information, was lowered from 0.12 to 0.06.

Based on these adjustments, ‘isw’ was calculated based on the following formula:

‘isw’ = (PSQ × 0.12 + SE × 0.6 + SE × 0.6 + TSI × 0.12 + EoS × 0.08 + EoO × 0.12 + LUM × 0.08) + (ETV × 0.10 + EUF × 0.08) + (SLQ × 0.12 + DIS × 0.06)(1)

Following this formula, the arrangement of weights for each ‘isw’ dimension is presented below:

‘isw’ = Urban Tissue variables × 64% + Urban Scene variables × 18% + Security × 18%(2)

**Figure 7:**
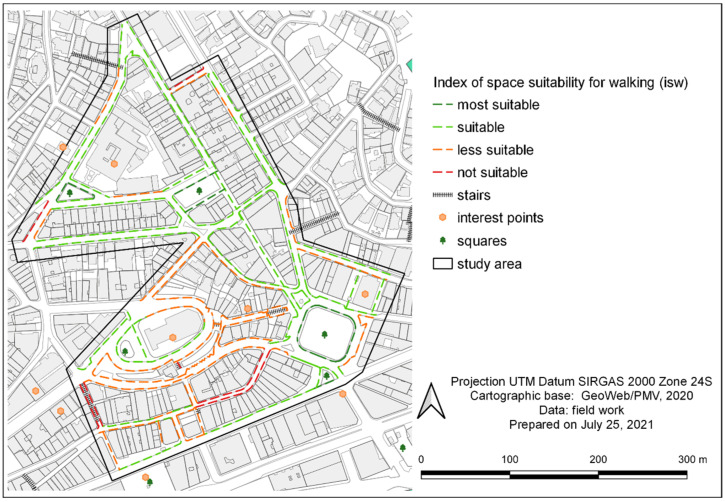
Map of the classification according to the walking space suitability index (‘isw’) in the study area.

The results of ‘isw’ calculation for each sidewalk stretch in the study area ranged from 1.24 (inadequate sidewalk) to 3.00 (most adequate sidewalk). Out of the 6706.5 m audited, 2660.1 m (39.7% of stretches) were evaluated as less adequate or inadequate for walking (Table 12). Using georeferencing by QGIS, this pedestrian network assessment has been mapped and can be more easily visualized in Figure 7.

In Figure 7, it can be observed that the study area presents a northern zone with better walkability and a southern zone with worse walkability. The latter has steeper slopes, little residential use, many closed commercial units, and abandoned sidewalks in a poor state of conservation.

### 4.3. The Index of Slopes and Stairs (‘is’)

The systematic review by [7] highlights that (i) sloping surfaces are a major walkability challenge for all ages, but especially for the older adults; (ii) few studies have investigated how sloping surfaces influence performance and modify walking patterns even for healthy older adults; studies were more interested in gait parameters and energy expenditure and little in the direct health benefits for the older adults; (iii) biomechanical studies use both ‘oxygen consumption’ and ‘heart rate’ as indicators of effort. 

The WIEH [7] uses heart rate (in bpm—beats per minute) as an indicator of effort and is based on studies that relate physical effort and inclination in public spaces. Based on its literature review and technical recommendations from Portugal, the WIEH considers <5% as adequate slope, from 5 to 8% as acceptable, and >8% as inappropriate. Because the WIEH links walking to the promotion of heart health in the older adults, physical effort should not exceed what is recommended as moderate activity (50–85% of maximum heart rate for the age group), nor should it exceed 120 bpm, the heart rate associated with an incline of up to 5%. Slopes above 8% would imply inadequate physical effort.

The ‘is’-index of slopes and stairs presents a combination of these factors to assess the suitability of a particular route or stretch. Based on the Brazilian NBR 9050 legislation, which deals with accessibility standards, the limit of 8.33% is used in this study as acceptable, which also meets the needs of people in wheelchairs (Table 13). The ‘is’ establishes the following classification parameters, relating inclination and heart rate.

Stairways were designated as such when possessing more than three steps. A ‘small number’ ranges from 4 to 8 steps, a ‘significant number’ from 9 to 16, and a ‘large number’ points to more than 16 steps. Each stretch was classified in terms of its slope and number of steps. In order to obtain the average slope of each sidewalk section, road slopes were evaluated according to altimetry data from the Alos Falsar Satellite and consequent digital elevation model, followed by cross-referencing data in QGIS with roads axes from GeoWeb. The evaluation of the number of steps made use of field survey data.

**Figure 8:**
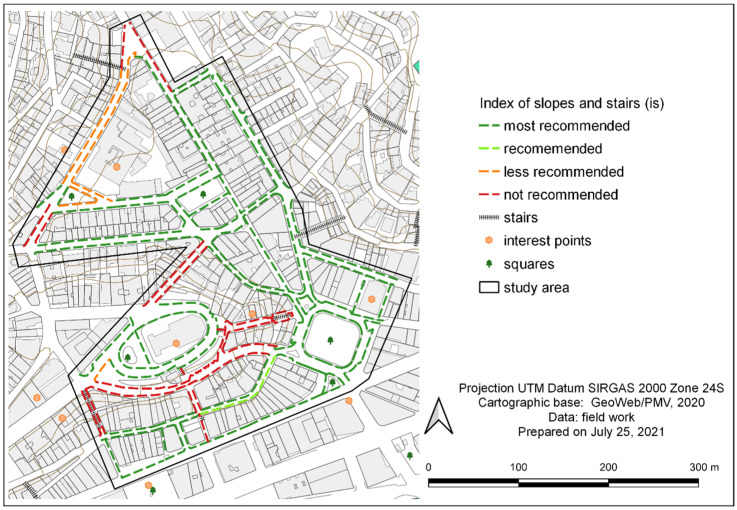
Map of stretch classifications within the study area, according to the index of slopes and stairs (‘is’).

Based on the parameters of ‘is’ presented in Table 13, the classification of the sidewalks in the study area (Table 14) pointed out that 4792.6 m are most recommended. However, 1328.3 m are not recommended, either because they had an excessive slope or because of the number of steps. With the use of QGIS georeferencing, this evaluation of slopes and stairways has been mapped in Figure 8.

Using the ‘isw’ and ‘is’ ratings, it was possible to proceed with the WIEH evaluation. 

### 4.4. The Walkability Index for Older Adults’ Health (WIEH)

The last step is the calculation of the WIEH based on the previous indexes-‘isw’ and ‘is’, according to the following formula:

WIEH = ‘isw’/is(3)

The WIEH evaluation (Table 15) demonstrates that 20.6% of sidewalk stretches within the study area were classified into the worst category, as ‘not age-friendly’ nor appropriate for older adults walking. Only 11.1% are ‘most age-friendly’. It is noteworthy that, for the sake of consistency and to avoid distortions, stretches considered inadequate by the ‘isw’ or not recommended by the ‘is’ kept the ‘non-recommended’ classification in the WIEH, since they are considered impediments to healthy walking by the older adults.

**Figure 9:**
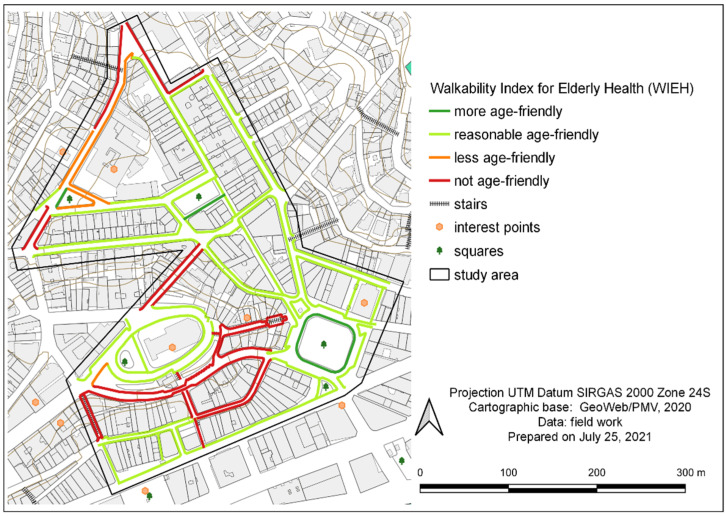
Classification of sidewalks according to the Walkability Index for Elderly Health (WIEH).

Figure 9 shows the spatialization of the WIEH data, allowing us to observe the location of the most age-friendly stretches of the sidewalk as well as of those with the worst conditions, requiring more attention from the municipality for inspection and correction of identified problems.

In Figure 9, a zone that concentrates not age-friendly sidewalks can be clearly observed, corresponding not only to areas with a steeper slope and/or with the presence of stairs (‘is’), but also to stretches where the sidewalks had already been poorly evaluated regarding the quality of the pedestrian network (‘isw’).

## 5. Discussion of Results

The comparison of pedestrian network for walking ratings (‘isw’) with the integration of slopes and stairs (‘is’), summarized in Table 16, suggests that the primary issue within the study area concerns mainly the quality of the pedestrian network itself—urban fabric, urban scene, and safety—and not exactly the slope. Of the stretches evaluated, 418.5 m were classified by the ‘isw’ as more suitable for the older adults to walk, while according to the ‘is’, 4792.6 m met the same classification.

In fact, due to their low inclinations, analyzed spaces do not present noticeable obstacles to older adults walking routes. As for stairways, there are five in the area and another six stretches with a significant number of steps; stairways thus constitute a more frequent occurrence than large inclines in the studied spaces.

**Figure 10:**
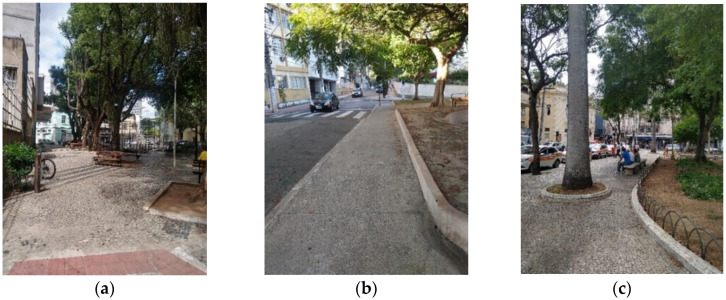
Examples of stretches with the best WIEH ratings, thus considered age-friendly routes. (**a**) Pedestrian walkway, next to the Ubaldo Ramalhete square; (**b**) Rua Coronel Monjardim, next to Praça Irmã Josefa Hosana and near the Senior Citizen Center; (**c**) sidewalk at Costa Pereira Square, a major city landmark, surrounded by the Carlos Gomes Theater and Glória Theater—it provides access to Cidade Alta and to tourist attractions such as the São Diogo Staircase and the Metropolitan Cathedral.

Figure 10a–c shows three of the stretches with the best WIEH evaluation, considered the friendliest to older adults’ walkability given their good physical conditions.

**Figure 11:**
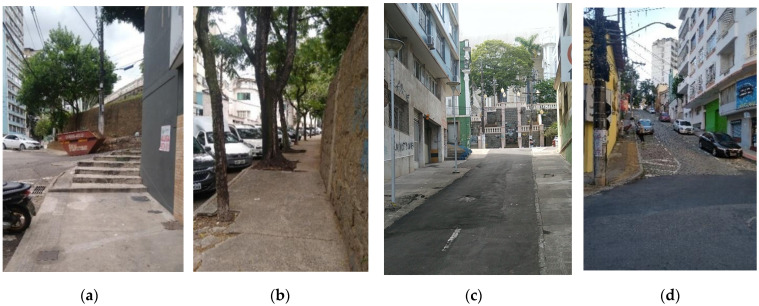
Examples of stretches with the worst classifications, and thus spaces unfriendly to older adults according to the application of the WIEH (**a**) Rua Dionísio Rosendo, with stairway at the inflection angle; (**b**) Rua Dionísio Rosendo, with narrowing of the passage and uneven floor, heightening the risk of falls; (**c**) Rua Cerqueira Lima, the only shared street in the study area, presents a steep slope and several potholes; in the background, the access stairway to the Cathedral, through Dionísio Rosendo Street; (**d**) Rua Prof. Baltazar, with an incline greater than 10%, and which also provides access to the Cathedral.

Figure 11 shows examples of sections with the worst WIEH scores and thus not considered age-friendly.

**Figure 12:**
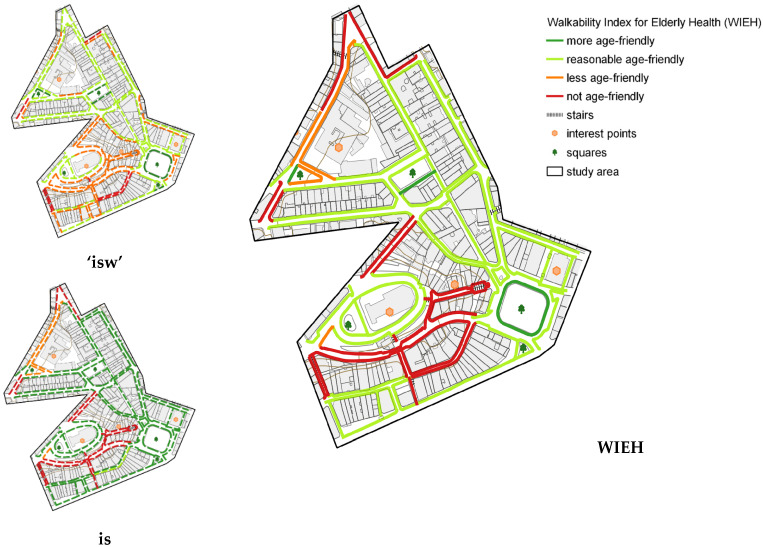
Comparison of ‘isw’, ‘is’, and WIEH ratings.

Figure 12 presents the comparison map of ‘isw’, ‘is’, and WIEH. The least age-friendly spaces, according to the WIEH, are mainly located in the area around the Metropolitan Cathedral (oval terrain), which was built on high ground. The stretches providing access to the Cathedral were classified as not age-friendly, given the steep slope, the presence of stairs, and/or the poor quality of the pedestrian network. The stretches south of the Cathedral present predominantly commercial use and several unoccupied buildings, in addition to abandoned sidewalks.

It should be noted that this comparison between ‘isw’ and ‘is’ maps with the WIEH (Figure 12) drew attention to a marked difference in the evaluation of some stretches-changing from ‘less adequate’ in the ‘isw’ (in orange) to ‘reasonably friendly’ to the older adults in the WIEH (in light green), which seems contradictory. This change in rating was induced by the WIEH parameters (Table 15), which were quite different from the ‘isw’ parameters (Table 12); more specifically, the broadening of the parameter for classification as ‘reasonably friendly’ in the WIEH, which covered values between >1.0 and ≤2.5. Observation of the ‘isw’, ‘is’, and WIEH maps in the study on Porto (Alves et al., 2020) had already suggested such a distortion, albeit not very pronounced.

**Figure 13:**
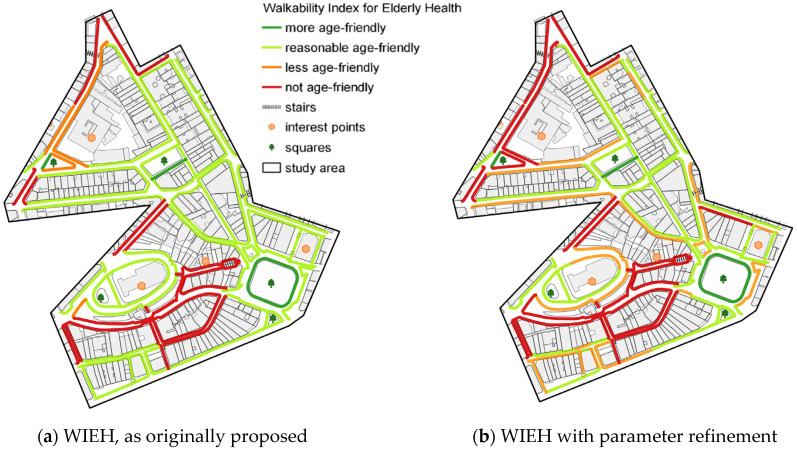
Comparison of WIEH ratings as per initial parameters in the previous study and the WIEH reclassifications in this study, through parameter refinement (**a**) WIEH as per Table 15 and Figure 9; (**b**) final WIEH, with the adjusted parameters, as per Table 17 (values of the ‘isw’ rating bands).

With this in mind and considering the weighting of the pedestrian network quality used in the ‘isw’ (Table 12), the same parameters were kept for the refinement of the WIEH (Table 17), leading to the new results shown in Figure 13. This adjustment increased the consistency of the WIEH result in relation to ‘isw’ and ‘is’. As a result, a change in the amount of ‘less friendly’ and ‘reasonably friendly’ stretches also occurred, as shown in Table 17 and Figure 13. Only 72 stretches, a little more than half (57.1%), display acceptable walking conditions, from ‘reasonably friendly’ (58 stretches) to ‘most friendly’ (14 stretches). The latter represent a small share of all stretches (11.1%). Finally, 28 stretches (22.2%) should be avoided.

Figure 13 demonstrates that the refinement of WIEH parameters proved adequate regarding its coherence. That said, unfortunately, the real walkability conditions for the older adults are not very adequate, as shown below.

The audit uncovered a diversity of road profiles, land use, and physical structures that, in general, present many obstacles to the walkability of older adults and their health. Several of the factors with the potential to impair the mobility of the older adults, as pointed out in the literature [52], were observed, revealing that the study area does not meet the requirements for an age-friendly city [6].

During the evaluation of the quality of the pedestrian network, the variables with the worst evaluation were street furniture for permanence (86% of stretches do possess any benches), diversity of information (71% of stretches do not have maps or direction signs for the pedestrian to orient themselves in the territory), existence of landscaping and arborization (65% of the stretches do not have any vegetation), and crossings (55% of the stretches with a pedestrian crossing of automobile spaces do not have ramps nor signage with the tactile floor). The absence of urban furniture to support walking and permanence in public spaces—especially the lack of benches, toilets, and drinking fountains—worsens the support conditions for walking. Even the 11 pedestrian-only streets (except for stairways) lack any supportive furniture, as already mentioned in Section 4. Of these streets, only one (9.1%)—in the stretch next to Praça Ubaldo Ramalhete—has benches, along with tables for playing chess and checkers. The remaining 10 pedestrian exclusive stretches (90.9%) not only lack benches, but some of them also lack paving maintenance.

It is worth noting that the inexistence of furniture to support the permanence of older adults in public spaces is a common problem in Brazilian cities, where public toilets and drinking fountains are very rare. Even benches are usually restricted to squares. There are plenty of bus stops in medium and large cities, but few have a shelter, which is often the only place to sit. This scarcity was highlighted by the fact that, in the study area, the few existing bus shelters were always full.

Another aspect of the study area that deserves to be mentioned is the scarcity of urban greenery, especially roadway afforestation, aggravating the discomfort of walking and staying in public space due to the microclimate conditions in the central urban area (urban heat island effect), namely the high summer temperatures and surface temperatures [41]. This issue is even more serious in wider roads, which are less shaded by buildings, especially considering the threat posed by extreme heat to older adults, since in addition to the pronounced discomfort, it increases the risk of heart attacks and strokes [42].

By visual and direct observation of the area, several of the problems highlighted in the literature [32] that can cause or aggravate the fear of falling and crossing the street by the older adults are also present, negatively affecting the route [52]. There are many inadequate crossings (76 crossings—60.3%), stretches with poor paving quality (39 stretches—31.0%), which can lead to falls and cause temporary or permanent injuries, and stretches with ‘acceptable’ paving but in need of requalification (46 stretches—36.5%). In general, a large number of potholes and irregularities were observed, scattered throughout different streets. Another aggravating factor for fall risk on sidewalks, which occurs in most Brazilian cities, is the lack of notifications.

Regarding the width of sidewalks, 18 stretches (14.3%) were observed to be too narrow, forcing pedestrians to leave the sidewalk and walk down the street, increasing the risk of being run over and killed; also noteworthy, 40 stretches (31.8%) do not meet the Brazilian accessibility standard [56] regarding the minimum width of 1.20 m. Among the 19 obstacles found in 15% of the stretches, which force the pedestrian to leave the sidewalk and circulate on the street, this study highlights the inappropriate location of streetlights on the sidewalks due to lack of free space, in contrast with the generous width of the motorized traffic lane.

When relating the result of the WIEH application in downtown Vitória, Brazil, to the published study on the application of the WIEH in downtown Porto, Portugal [13], the influence of the characteristics of the pedestrian network (‘isw’) and of the slopes and stairways (‘is’) over the final composition of the index is worth mentioning. In Porto, the least favorable conditions are related to the topography and therefore expressed in the integration of slopes and staircases (‘is’)—an aspect that is challenging for any municipality to solve. In Vitória, the less favorable conditions pertain to the quality of the pedestrian network (‘isw’), a more easily avoidable problem.

A visit to the city of Porto during the research that led to this article allowed us to observe in loco the good quality paving of sidewalks and crossings, and the fact that potholes are very rare—a situation opposite to the study area in Vitória, where there is rarely a block without potholes or unevenness and few safe crossings. One factor that may explain this great disparity is precisely the sphere of responsibility for the execution and maintenance of sidewalks in each city, while in Porto, the responsibility lies with the municipality, in Vitória—as in most Brazilian municipalities—it lies with the owners of the lots along a block, leading to a great diversity of pavements and maintenance. This contradiction of delegating responsibility for public space to the private individual is at the root of the great variation in the quality and frequent poor condition of sidewalks in Brazilian cities [48,49,50]. Another aspect that drew attention in Vitória and Porto—and not only in the study areas—was the great disparity in the provision of street furniture, a disparity also observed in the results of the WIEH application, and even more so during the visit to Porto. In this case, the responsibility for the provision of adequate street furniture in both cities lies with the municipality, and thus the cause of such disparity is not immediately apparent.

Many of the problems identified in this study can be easily reversed, for example, through small repairs to the sidewalks. The pedestrian-only streets and some squares could be prioritized in the provision of furniture, such as benches, flower boxes, drinking fountains, and even toilets. Planting trees is possible even on streets with narrower sidewalks: it is common for parking lanes to run along the street, and thus trees can be interspersed between parking spaces; furthermore, removing some spaces can improve the vehicle-laden landscape of the central area. The largest challenge lies in obstacles placed on sidewalks, such as power poles (Duque de Caxias, Treze de Maio, and Basílio Daemon streets). Each location is a specific case, with possible solutions including (i) narrowing the vehicle lane or removing parking lots to widen the sidewalk or painting a pedestrian lane on the asphalt next to the sidewalk; (ii) transforming a segregated street into a shared one; (iii) restricting vehicle access, allowing only residents to circulate.

Streets with steeper slopes, even though they are not the most recommended for walking, also need adjustments such as anti-slip flooring, handrails in strategic areas, and, in extreme cases, the possible deployment of mechanized aids for overcoming the inclination (elevators, escalators, funiculars). A resident who approached us during the field survey drew attention to the great difficulty of climbing Professor Baltazar Street (Figure 11d), often used by the older adults to reach the Cathedral. This street is 80 m long and has a slope of more than 10%, requiring great effort. During the field survey, there was an instance of a lady walking up the street leaning against a store wall to rest. This street is quite wide, with parking lanes on both sides, and could accommodate a lane for a mechanical aid or at least for flat resting spaces with benches. The total absence of public toilets in the study area cannot be overlooked, and there is no lack of space for them, given the many squares and pedestrian streets.

The application of the WIEH in the study area of Vitória met the goal of assessing the walking conditions of older adults and indicating the most suitable routes for health purposes, as well as those to be avoided. Making known the most suitable sidewalks and routes for older adults’ walkability encourages walking along more friendly routes, thus leading to more comfortable and frequent walks. From an urban planning perspective, once the problems in each dimension of the index are identified and spatially located, one can proceed towards specific repair actions and, eventually, more comprehensive actions such as road remodeling to prioritize walkability, review of parking lot supply, implementation of street tree planting, stimulus to occupy idle buildings, among other actions that can requalify downtown Vitória in a specific or more structural way. In the case of upgrading projects, it is important for the municipality to consult the population, especially the older adults, to jointly define the priority areas for intervention, furnishings, and other desired aspects.

In the study area, several aspects that discourage walking were observed, especially for the older adults, but also for other groups with mobility restrictions such as small children, pregnant women, obese people, or parents with strollers. Although downtown Vitoria is often called ‘a walker’s paradise’ by WalkScore [56,57] since, in fact, one does not need to use a car for daily activities, the area imposes enormous constraints upon the walkability of older adults and their health due to the poor quality of its pedestrian network.

## 6. Conclusions

The application of the WIEH in the historic center of the Brazilian city of Vitória-ES and the refinement of its classification parameters allowed for an evaluation of the conditions for older adults and healthy walking, as well as a validation of the index. The evaluation of the variables in a disaggregated manner created an opportunity to measure the contribution of each variable to the index. In the studied area, the predominantly flat topography favors walking, even though there are some zones with steeper slopes. However, critical factors were identified regarding the poor quality of the pedestrian network, a fact that corroborates studies on walkability in Brazilian cities. The few really age-friendly sidewalks are located next to squares and are the responsibility of public authorities.

The application of the WIEH in Vitória has proven useful as a support tool for the municipality in monitoring the factors that affect the walkability of older adults, as well as for aiding the urban planning and design sectors in making improvements in the area. It is also useful for older adult users because the WIEH map can help them choose the best routes for walking, accessing urban services and places of historical interest. In this sense, the research findings can also be used by the residents’ association, the Elderly Community Center, and the Municipal Council of the Elderly (Conselho Municipal do Idoso-Comid), which collaborates with the formulation of policies, programs, and projects to improve the quality of life of older adults in Vitória.

Limitations of the methodology used lie mostly with the fact that it requires a very detailed field survey, which makes its application in large areas difficult, although the survey of the WIEH itself is relatively simple. One way to get around this limitation is to delimit areas of priority or of greatest interest, for example, by selecting neighborhoods with a higher percentage of older adults, the concentration of priority activities, or consultation opportunities with the population. Another limiting aspect concerns the evaluation of road slopes: these data were not available in the municipal database and obtaining the average slope values for each road required several geoprocessing procedures. Slope average may not be the most satisfactory figure for cases in which there is great variation in topography, especially if the slope is not regular, as in some streets within the study area. However, it is possible to survey the slope in each lane with one of many mobile phone apps.

The general implementation of the methodology used is quite simple and easy to apply in any city, all that is required is to delimit a territorial clipping and follow the methodological steps set out in this article: physical audit with the aid of a printed form and photographic record; assessment of the quality of the pedestrian network (isw); assessment of slopes and stairways (is), and determination of the friendliest routes for older adults (wieh).

It is strongly recommended that future research—in European or Brazilian contexts—use the review of the WIEH values proposed in this paper. For Brazilian cities, it is also recommended to use the weights and variables as proposed in this paper, which is already adapted to the Brazilian context, especially regarding the accessibility standard.

In the Brazilian context, in addition to this refinement of WIEH, we also recommend a greater gradation in the evaluation of the variables that make up the pedestrian network, going from three to four or five levels, in order to better reflect nuances. Given the strong Brazilian inequalities, from the regional to the intra-urban scales, it is important to have comparative studies (especially within different areas in the same municipality) that evidence and quantify inequalities pertaining to the walkability of older adults, with a view to providing support for problem reversal in the urban structure. A relevant aspect is to complement the applications of the WIEH with qualitative-based research, to survey the perception of older adults, and even to test the age-friendly paths themselves in order to validate the method or determine adjustments.

For future research, we hope to be able to carry out a qualitative survey with older adults as well as carry out new surveys in other areas in the city of Vitoria, Brazil.

The present study thus contributes to the field of research on walkability by working with an index that fills existing gaps—the WIEH incorporates inclines and stairways into the physical audit and relates walkability conditions to the potential for direct health benefits for older adults. In addition to this scientific contribution, studies like this application of the WIEH can shed light on the discussion of older adults’ walkability, mobilize groups, guide public policies, and guide interventions to adapt cities to active and healthy aging. An age-friendly city is welcoming for everyone.

## Figures and Tables

**Table 1:** Dimensions and related urban variables of the WIEH.

Dimensions	Variables
Urban fabric	Surface/floor quality; existence and width of sidewalk; intersections/crossings; existence of stairs/steps; presence of obstacles; mixed use
2. Urban scene	Existence of trees/vegetation; existence of street furniture
3. Safety	Quality of street lighting; diversity of information

Source: Walkability Index for Elderly Health: A Proposal [7].

**Table 2:** Pedestrian surface quality (PSQ).

PSQ Evaluation	Nr. of Stretches	%
1—poor	39	31.0
2—acceptable	46	36.5
3—good	41	32.5

**Table 3:** Sidewalk width (SW).

SW Evaluation	Width	Nr. of Stretches	%
1	SW < 0.90 m	18	14.3
1.5	0.90 ≤ SW < 1.20 m	22	17.5
2	1.20 ≤ SW < 1.5 m	24	19.0
2.5	1.50 ≤ SW < 1.80 m	20	15.9
3	SW ≥ 1.8 m	42	33.3

**Table 4:** Traffic street intersection (TSI).

TSI Evaluation	Nr. of Stretches	%
1—inadequate crossing (lack of ramp and tactile floor at both ends)	47	37.3
2—adequate slope at one end only	29	23.0
3—adequate crossing (no crossing issues)	50	39.7

**Table 5:** Existence of stairs (EoS).

EoS Evaluation	Nr. of Stretches	%
1—3 flights of stairs or more	5	4.0
2—1–2 flights of stairs	6	4.8
3—no stairs in this stretch	115	91.3

**Table 6:** Existence of obstacles (EoO).

EoO Evaluation	Nr. of Stretches	%
1—systematically affect walking	19	15.1
2—occasionally affect walking	49	38.9
3—no obstacles throughout the stretch	58	46.0

**Table 7:** Land use mix (LUM).

LUM Evaluation	Nr. of Stretches	%
1—no mixed use within the stretch	45	35.7
2—medium mixed use (at least 2 different uses)	34	27.0
3—high mixed use	47	37.3

**Table 8:** Existence of trees/vegetation (ETV).

ETV Evaluation	Nr. of Stretches	%
1—no trees/vegetation	82	65.0
2—moderate presence of trees/vegetation	22	17.5
3—strong presence of trees/vegetation	22	17.5

**Table 9:** Existence of urban furniture (EUF).

EUF Evaluation	Nr. of Stretches	%
1—no urban furniture	101	86.3
2—moderate presence of urban furniture	0	-
3—strong presence of urban furniture	16	13.7

**Table 10:** Street light quality (SLQ).

SLQ Evaluation	Nr. of Stretches	%
1—low	12	9.5
2—acceptable	49	38.9
3—good	65	51.6

**Table 11:** Diversity of information signs (DIS).

DIS Evaluation	Nr. of Stretches	%
1—low (lack of information)	90	71.4
2—medium	19	15.1
3—high	17	13.5

**Table 12:** The Index of Space Walkability (‘isw’) within the study area.

Value	Classification	Nr. of Stretches	%	Length (m)	%
1–1.49	Inadequate	6	4.8	449.0	6.7
1.5–1.99	Less adequate	40	31.7	2211.1	33.0
2–2.49	Adequate	67	53.2	3627.9	54.1
2.5–3.0	Most adequate	13	10.3	418.5	6.2

**Table 13:** Parameters for the Index of Slopes and Stairs (‘is’) within the study area.

Value	Slope	Stairway	Description
1	Adequate (<5%)	No stairway	Most recommended
2	Adequate (<5%)	Small number of steps	Recommended
3	Acceptable (5–8.33%)	Significant number of steps	Less recommended
4	Steep (>8.33%)	Great number of steps	Not recommended

**Table 14:** The Index of Slopes and Stairs (is) applies to the study area.

‘is’ Classification	Nr. of Stretches	%	Length (m)	%
1—most recommended	96	76.2	4792.6	71.5
2—recommended	1	0.8	115.1	1.7
3—less recommended	7	5.6	470.4	7.0
4—not recommended	22	17.5	1328.3	19.8

**Table 15:** Walkability Index for Elderly Health (WIEH).

Value *	Description **	Observation	Nr.	%
≤0.5	Not age-friendly	Non-recommended stretches	26	20.6
>0.5 and ≤1.0	Less age-friendly	Stretches better avoided	6	4.8
>1.0 and ≤2.5	Reasonably age-friendly	Stretches to be considered	80	63.5
>2.5 and ≤3.0	Most age-friendly	Highly recommended stretches	14	11.1

*, ** in accordance with Walkability Index for Elderly Health: A Proposal [7].

**Table 16:** Comparison between ‘isw’ and ‘is’.

Evaluation	‘isw’ (m)	is (m)	‘isw’ (% Stretches)	is (% Stretches)
Inadequate	449.0	1328.3	4.8	17.5
Less adequate	2211.1	470.4	31.7	5.6
Adequate	3627.9	115.1	53.2	0.8
More adequate	418.5	4792.6	10.3	76.2

**Table 17:** Review of the Walkability Index for Elderly Health (WIEH) values and outcome in the study area.

Value *	Description	Observation	No.	%	Length (m)	%
≤1.49	Not age-friendly	Non-recommended stretches	26	20.6	2065.0	30.8
>1.49 and ≤1.99	Less age-friendly	Stretches better avoided	28	22.2	969.7	14.5
>1.99 and ≤2.50	Reasonably age-friendly	Stretches to be considered	58	46.0	3182.3	47.5
>2.50 and ≤3.00	Most age-friendly	Highly recommended stretches	14	11.1	489.5	7.3

* values readjusted to the ‘isw’ rating bands.

## Data Availability

This study used as a basis a publicly available sociodemographic and spatial dataset. This data can be found here: https://geoweb.vitoria.es.gov.br/.
